# A retrospective review of the Pediatric Development Clinic implementation: a model to improve medical, nutritional and developmental outcomes of at-risk under-five children in rural Rwanda

**DOI:** 10.1186/s40748-017-0052-2

**Published:** 2017-07-12

**Authors:** Eric Ngabireyimana, Christine Mutaganzwa, Catherine M. Kirk, Ann C. Miller, Kim Wilson, Evodia Dushimimana, Olivier Bigirumwami, Evelyne S. Mukakabano, Fulgence Nkikabahizi, Hema Magge

**Affiliations:** 1 Rwinkwavu District Hospital, Ministry of Health, Rwinkwavu, Rwanda; 2 Department of Pediatrics, Partners In Health/Inshuti Mu Buzima, Rwinkwavu, Rwanda; 3 Division of Global Health Equity, Brigham and Women’s Hospital, Boston, USA; 4 Division of General Pediatrics, Boston Children’s Hospital, Boston, USA; 5 Harvard Medical School, Department of Global Health and Social Medicine, Boston, USA; 6 Division of Clinical Services, Ministry of Health, Kigali, Rwanda; 7 P.O. Box 3432, Kigali, Rwanda

**Keywords:** High-risk newborns, survival, early childhood development, primary care, sub-Saharan Africa

## Abstract

**Background:**

As more high-risk newborns survive the neonatal period, they remain at significant medical, nutritional, and developmental risk. However, no follow-up system for early intervention exists in most developing countries. In 2014, a novel Pediatric Development Clinic (PDC) was implemented to provide comprehensive follow-up to at-risk under-five children, led by nurses and social workers in a district hospital and surrounding health centers in rural Rwanda.

**Methods:**

At each PDC visit, children undergo clinical/nutritional assessment and caregivers participate in counseling sessions. Social assessments identify families needing additional social support. Developmental assessment is completed using Ages and Stages Questionnaires. A retrospective medical record review was conducted to evaluate the first 24 months of PDC implementation for patients enrolled between April 2014–December 2015 in rural Rwanda. Demographic and clinical characteristics of patients and their caregivers were described using frequencies and proportions. Completion of different core components of PDC visits were compared overtime using Fisher’s Exact test and *p*-values calculated using trend analysis.

**Results:**

426 patients enrolled at 5 PDC sites. 54% were female, 44% were neonates and 35% were under 6 months at enrollment. Most frequent referral reasons were prematurity/low birth weight (63%) and hypoxic-ischemic encephalopathy (34%). In 24 months, 2787 PDC visits were conducted. Nurses consistently completed anthropometric measurements (age, weight, height) at all visits. Some visit components were inconsistently recorded, including adjusted age (*p* = 0.003), interval growth, danger sign assessment, and feeding difficulties (*p* < 0.001). Completion of other visit components, such as child development counseling and play/stimulation activities, were low but improved with time (*p* < 0.001).

**Conclusions:**

It is feasible to implement PDCs with non-specialized providers in rural settings as we were able to enroll a diverse group of high-risk infants. We are seeing an improvement in services offered at PDCs over time and continuous quality improvement efforts are underway to strengthen current gaps. Future studies looking at the outcomes of the children benefiting from the PDC program are underway.

## Background

Global advances have been made over the past decade to improve neonatal care and the burden of neonatal mortality and morbidity in low and middle-income countries [1] with prematurity as the leading cause of death among children under 5 years of age [2]. Ongoing efforts to eliminate preventable neonatal deaths globally must also be accompanied by efforts to ensure children not only survive but also thrive [3]. The first 3 years of life are a critical period of brain development [4]; interventions during this early period can improve growth and development [5] which impact long-term outcomes including educational attainment and productivity later in life [6, 7]. Certain populations of children are at increased risk of developmental delay and growth faltering, such as those born preterm and low birth weight, or with hypoxic-ischemic encephalopathy (HIE), congenital malformations, genetic syndromes, or birth injuries. It is known that these high-risk infants can have a range of challenges such as vision and hearing impairment, speech and motor delays, and poor nutrition, among others [8–11]. In high-income countries, longitudinal follow-up of these children is integrated into routine pediatric care and is typically conducted by specialists [12]. In sub-Saharan Africa, there are very few interventions to support early childhood development and the majority are generalized, community-based programs [13, 14] that do not meet the unique needs of high-risk infants. The use of an intensive home-visiting model has shown promise for improving the development of children with HIE in low and middle income countries [15]. However, to our knowledge there are no early childhood development interventions integrated into routine primary care for at-risk children in low-income countries without access to pediatricians and subspecialists, especially in sub-Saharan Africa.

Rwanda has made tremendous improvements in reducing under-five mortality [16], and expanded access to neonatal care through neonatal units at district hospitals and essential neonatal care at health centers. However, there is no formal follow-up system for children after being discharged from hospital neonatal units, especially in rural areas where there are no specialists; these children remain at increased risk for mortality and morbidities into childhood [17]. “A follow-up of preterm and low birth weight children discharged from Rwinkwavu District Hospital neonatal unit between October 2011 and October 2013 showed rates of chronic and acute malnutrition that were double and triple national prevalence rates, respectively, and that caregivers frequently reported feeding difficulties. Furthermore, two-thirds of the children had an abnormal developmental screening (Kirk, et al., unpublished data)".

In Rwanda, screening for malnutrition in infants and young children is conducted at the community level by community health workers [18] and early childhood development programs have been piloted in selected communities [19, 20]. As in many other sub-Saharan African countries, a barrier to delivering specialized follow-up is the limited availability of pediatricians outside of higher-level referral facilities [21], and other specialists such as developmental pediatricians, neurologists, and physical, speech and occupational therapists. Task-shifting has been used as an approach across low-resource settings to overcome the gap between the availability of specialized providers and service needs [22]. However, this approach has not been explored for pediatric follow-up care among high-risk infants.

Since April 2014 the Ministry of Health, in collaboration with Partners In Health/Inshuti Mu Buzima and UNICEF, has been implementing the Pediatric Development Clinic (PDC) to serve neonates discharged from a rural hospital neonatal unit and other high-risk children under-five in need of follow-up. The PDC provides medical, nutritional and developmentally supportive programs to allow for early identification of complications and intervention to support children to reach their full potential. We hope that this study will provide a pragmatic approach to implementing a feasible clinic model for comprehensive medical, nutritional, developmental support for high-risk infants until age 5 years in resource-limited settings without access to complex care services and medical specialists.

## Methods

### Study aim and design

This study aims to provide a description of the PDC intervention’s first 2 years of implementation using a retrospective electronic medical record review of routinely-collected data. The study population includes all children referred into PDC from April 2014–December 2015. In order to ensure all referred children completed at least one visit following their referral date, we analyzed all of the visits for these children in the first 2 years of clinic implementation (April 2014–March 2016). We hypothesize that as the program matures, over time we will see greater adherence to clinic procedures and increase in documentation of appropriate clinical and developmental assessment and follow-up.

### Study intervention

**Fig. 1:**
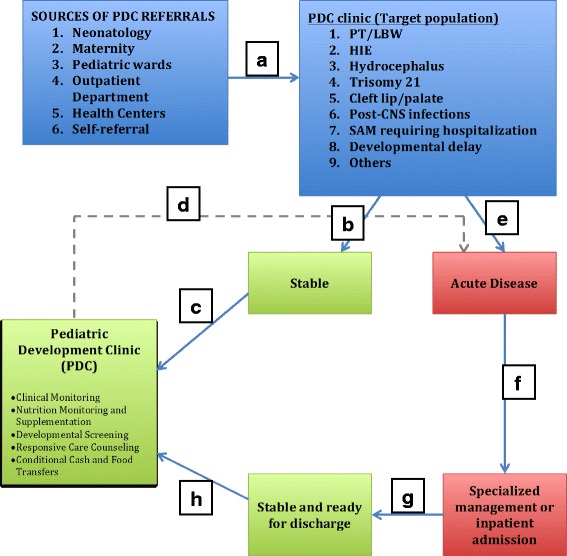
Pediatric Development Clinic (PDC) Referral and Clinic Flow. Routine Clinic Flow: Different health facilities and services (**a**) refer all stable under-five children meeting referral criteria (**b**) to PDC for enrolment (**c**). At any PDC visit assessment, if an acute illness is identified, an immediately referral (**d**) for acute care is issued (**e**) followed by specialized care (**f**) if needed. When the child has been stabilized and preparing for hospital discharge (**g**), a referral back to continue PDC follow-up (**h**) is completed

This study was conducted in the Rwinkwavu District Hospital catchment area in Southern Kayonza District in rural Rwanda. Rwinkwavu District Hospital is a public Ministry of Health facility supported by Partners In Health/Inshuti Mu Buzima since 2005. Southern Kayonza serves a population of approximately 188,363 [23] with one district hospital and eight health centers. Rwinkwavu District Hospital has provided neonatal care since 2010 and was among the first district hospitals to initiate specialized neonatal care services [24]. The first PDC was opened in April 2014 at Rwinkwavu District Hospital to provide medical, nutritional and developmental follow-up for high-risk children discharged from the neonatal unit (see Fig. 1). Four months later, in August 2014, PDC was expanded to two health centers and thereafter to two more in June 2015.

The PDC serves children with prematurity, birth weight under 2000 g, HIE, hydrocephalus, cleft lip and palate, trisomy 21, or other developmental delays. In April 2015, additional referral criteria – post-central nervous system infections (meningitis and cerebral malaria) – were added, and in February 2016 (after the inclusion period for this study), children diagnosed with severe acute malnutrition requiring hospitalization began being referred to PDC after discharge from the hospital. Children are referred to PDC using standardized referral criteria and a form completed from Rwinkwavu District Hospital neonatal unit, maternity ward, pediatric ward or emergency department when a child meeting referral criteria is identified. The goal is to identify at-risk infants as young as possible for early enrollment. Children may additionally be referred informally from outpatient clinics at health centers and Rwinkwavu District Hospital, health center maternity wards or via self-referral from the community.

The clinic was designed to be integrated into the public system with as minimal additional external resources as possible to maximize the potential for scalability. Building on an existing platform for chronic care services, the PDCs were integrated into the non-communicable disease (NCD) clinics in the public health system. Only one additional staff member was hired at the district hospital clinic due to the addition of patients to an already high-volume NCD clinic. The PDC is operated at each site on a weekly basis. The PDC team is led by a general practitioner based at the Rwinkwavu District Hospital PDC with routine clinic visits conducted by a nurse and social worker. Each clinic is equipped with a child-friendly space for group counseling and play, and a consultation room with basic furnishings, medical equipment for assessing growth and vitals, as well as structured medical record forms for monitoring patients. Health center teams receive mentorship and supervision from the district hospital team, and the hospital team receives mentorship from additional technical advisors as needed (including a pediatrician and a nutritionist). Prior to initiation of PDC, each team was trained on the PDC protocol and completion of medical record forms for each specific condition. When a health center clinic is first opened, the district hospital doctor, nurse and social worker provide weekly mentorship to provide intensive support following the initial training. After approximately two to 3 months of weekly mentorship (and depending on assessed level of skill and comfort of the providers) mentorship de-intensifies to at least one visit per month. The supervision team meets monthly to discuss strengths and weaknesses of implementation at each clinic, address specific challenges, and create future plans for the clinic.

On a visit day, children’s clinical, nutritional and developmental status are assessed by the nurse. According to the protocol, the following are documented for all children: age, weight, height, interval growth for infants up to 6 months, head circumference for infants up to 18 months, immunization status, and vital signs. The child’s anthropometric measurements are plotted on World Health Organization growth charts [25, 26] and counseling provided accordingly. Also, a condition-specific protocol of additional screening is completed based on the reason for referral. For preterm infants – the primary referral base – this includes Kangaroo Mother Care (KMC) follow-up, micronutrient supplementation, and vision/hearing screening among other standard follow-up measures. Developmental screening using an Ages and Stages Questionnaire (ASQ-3) [27] is conducted at ages 6, 12, 24, 36, 48 and 60 months; the ASQ-3 was previously adapted and translated into the local language, Kinyarwanda, for use in Rwanda [19].

In addition, the children’s primary caretakers participate in group education led by the social worker on different topics depending on the age of their children such as KMC, hygiene, nutrition, family planning, breastfeeding, and the importance of play and early stimulation. A structured social assessment is performed for further individual counseling and to identify families who qualify for conditional food or cash transfers, or additional counseling and home visits according to pre-determined clinical and social criteria. Rwanda’s community-based poverty categorizations, known as *Ubudehe*, were used to establish social need [28]; *Ubudehe* is a government mechanism to help communities identify the most vulnerable households so that government-provided social support to achieve poverty reduction can be targeted to these households. Home visits are conducted weekly by social workers with nurses to provide support to families identified during clinic and social assessments as needing additional or home-based support.

### Data collection

Data were extracted from electronic medical records (EMR) and paper-based patient charts by trained data collectors. The EMR undergoes routine data quality checks, and discrepancies for key indicators between paper-based charts and the EMR were addressed during data collection.

### Measures

Demographic characteristics for children and their primary caretakers were extracted from patients’ referral and intake forms. Children’s age was categorized as neonates less than 1 month, infants 1 to 5 months, 6 to 11 months and children 12 months and above. Gestational age in weeks was categorized according to WHO classification as “extremely preterm” or less than 28 weeks, “very preterm” from 28 to less than 32 weeks, “moderate to late preterm” from 32 to less than 37 weeks and “term” as 37 weeks and above. Birth weight in grams was categorized as “extremely low birth weight” (less than 1000 g), “very low birth weight” (from 1000 to 1499 g), “low birth weight” (1500 to 1999 g) and birth weight above 2000 g. Mother’s age was categorized in intervals from 15 to less than 20, from 20 to 24, 25 to 34 and 35 to 44 years and 45 years and above. Caretakers’ socio-economic status was categorized using Rwanda’s *Ubudehe* classification based on households that qualify for automatic government support or those who do not qualify. The number of other dependents in a household was categorized as “none” if the child attending PDC was the only child in the home, or “1–3”, “4 to 5”, or “6 and above”.

Based on visit forms that were completed at each patient visit, we assessed whether the nurse had conducted the required assessments at each patient visit according to the PDC protocol. To assess changes in clinic implementation over time, patient visits were grouped into 6 months intervals: April to Sept 2014, October 2014 to March 2015, April to September 2015 and October 2015 to March 2016.

### Analysis

We described patient demographic and clinical characteristics, and programmatic process using frequencies and proportions for categorical variables. We compared percent of different aspects of PDC protocol completed at each visit across 6 months intervals using a non-parametric test for trend [29] to determine if there were significant differences in protocol adherence over time.

### Ethics

The Rwanda National Ethics Committee (RNEC) No.882/RNEC/2016 and Ministry of Health approved this study. Additionally, the institutional review board at Boston Children’s Hospital (Boston, Massachusetts) exempted this study from review.

## Results

From April 2014 to December 2015, 426 children enrolled in the PDC. Table 1 describes characteristics of the participants and their caregivers. Of 417 children with gender recorded, more than half were female (54.2%, *n* = 225/417). Most were neonates (43.7%, *n* = 172/394) or less than 6 months old (35.0%, *n* = 138/394) at the time of enrollment. Most enrollees (56.7%, *n* = 160/282) were preterm. The clinic also enrolled children with a birth weight under 2000 g, with 53.5% (*n* = 124/334) of those with available birth weight data in the low, very low or extremely low birthweight categories. Most enrolled children were born at Rwinkwavu District Hospital (64.7%, *n* = 247/382) and were referred into PDC from hospital departments. Two-thirds of children (64.3%, *n* = 214/333) were living in households with one to three other dependents.

**Table 1:** Demographic characteristics of children enrolled in PDCs from April 2014 to March 2016 and their caretakers

Child characteristics	Total *N* = 426	
	n	%
Gender (*N* = 415)		
Male	190	45.8
Female	225	54.2
Age at first visit (*N* = 394)		
Neonate (< 1 month)	172	43.7
1–5 months	138	35.0
6–11 months	26	6.6
12 months and above	58	14.7
Gestational age in weeks (*N* = 282)		
Extremely preterm (<28 weeks)	13	4.6
Very preterm (29 to <32 weeks)	38	13.5
Moderate to late preterm (32 to <37 weeks)	109	38.7
Term (37 + weeks)	122	43.3
Birth weight categories (grams) (*N* = 334)		
Extremely low, < 1000 g	4	1.2
Very low, 1000–1499 g	61	18.3
Low, 1500–1999 g	114	34.1
2000 g or more	155	46.4
Site of birth (*N* = 382)		
District Hospital	247	64.7
Health Center	97	25.4
Home	26	6.8
En route	8	2.1
National Referral Hospital	4	1.1
Referral site (*N* = 355)		
District Hospital	310	87.3
Health Center	24	6.8
Other	21	5.9
Caretaker characteristics	n	%
Relationship with the child (*N* = 312)		
Mother	306	98.1
Father	2	0.6
Grandmother	3	1.0
Other relative	1	0.3
Mother’s age (*N* = 292)		
15–19 years	17	5.8
20–24 years	98	33.6
25–34 years	120	41.1
35–44 years	52	17.8
45 years and above	5	1.7
Marital status (*N* = 383)		
Married or cohabitating	337	88.0
Not currently married/ cohabitating	46	12.0
HIV status (*N* = 367)		
Positive	23	6.3
Negative	344	93.7
Caretaker education level (*N* = 343)		
No formal education	57	16.6
Some primary	142	41.4
Primary completed	128	37.3
Secondary or higher completed	16	4.7
*Ubudehe* classification (*N* = 282)		
Does not qualify for government support	202	71.4
Qualifies for government support	80	28.3
Household lives within Rwinkwavu catchment area (*n* = 413)		
Yes	383	92.7
No	30	7.3
Transport means to the health facility (*N* = 408)		
Walking	177	43.4
Bicycle	4	1.0
Motorbike	149	36.5
Mini bus	54	13.2
Other	24	5.9
Number of dependents in a household (*N* = 333)		
No other children	23	6.9
1 to 3	214	64.3
4 to 5	66	19.8
6 and above	30	9.0

Almost all children were brought to the PDC by their mothers (98.1%, *n* = 306/312). The majority of caregivers were between 25 and 34 years of age (41.1%, *n* = 120/292), and were married or cohabitating (88%, *n* = 337/383), and had some primary school education (41.4%, *n* = 142/343). Nearly all patients were living in Rwinkwavu catchment area (92.7%, *n* = 383/413) with some patients coming from neighboring hospital catchment areas. Of caregivers, 6.3% (*n* = 23/367) were known to be living with HIV. The most common mode of transport to the clinic was walking (43.4%, *n* = 177/408) or motorcycle taxis (36.5%, *n* = 149/408). Of those with *Ubudehe* status recorded, 71.4% (*n* = 202/282) percent did not qualify for automatic government support.

The most frequent diagnosis of PDC clinic patients was prematurity and/or low birth weight (62.9%, *n* = 266/423) followed by HIE (33.7%, *n* = 142/422, Table 2). Less common diagnoses included hydrocephalus (1.3%, *n* = 5/390), trisomy 21 (2.3%, *n* = 9/390), and cleft lip and/or palate (1.8%, *n* = 7/390). Fifty-six children had more than one diagnosis (13.2%).

**Table 2:** Medical diagnoses of PDC patients at enrollment in Rwinkwavu catchment area

Medical conditions	n	%
Children with any diagnosis on record		
Diagnosed as preterm/LBW (*N* = 423)	266	62.9
Diagnosed with HIE (*N* = 422)	142	33.7
Diagnosed with Hydrocephalus (*N* = 390)	5	1.3
Diagnosed with Trisomy 21 (*N* = 390)	9	2.3
Diagnosed with Cleft lip and/or palate (*N* = 390)	7	1.8
Diagnosed with other development delays (*N* = 390)	22	5.6
Diagnosed with other condition (*N* = 390)	22	5.6
Child has multiple diagnoses (*N* = 423)	56	13.2

We assessed whether families had received social support and developmental screening with the ASQ-3 according to protocol (Table 3). Of 364 participants with social support data available, almost every mother received transport fees at one or more visits during PDC (96.7%, *n* = 352) and 43.7% received food packages (*n* = 159). Additionally, more than one-third of children with available data (36.3%, *n* = 132) received infant formula for some period of time as part of a treatment plan for insufficient maternal breastmilk supply or other difficulties with breastfeeding to achieve adequate growth. Developmental screening using the ASQ-3 was completed for more than the half of children in the clinic who were 6 months or older (56.4%, *n* = 184/326 eligible children 6 months or older).

**Table 3:** Developmental screening and social support provided to PDC patients at enrolment

	n	%
Socioeconomic assistance ever received (*N* = 364)		
Transport fees	352	96.7
Infant formula	132	36.3
Food package	159	43.7
ASQ assessment completed if child is 6+ months old (*N* = 326)		
Yes	184	56.4
No	142	43.6

The proportion of visits over time with clinical, nutritional and developmental screening and counseling completed according to protocol are shown in Table 4. Overall, age, weight, height/length, and head circumference were measured consistently at visits (98.3%, *n* = 2486/2529; 98.9%, *n* = 2755/2787; 98.2%, *n* = 2736/2787 and 95.8%, *n* = 2366/2431 respectively). Vaccination status was assessed at 88.1% (*n* = 2138/2529) of visits and feeding difficulties were assessed at 84.5% (*n* = 2138/2529) of visits. Danger signs were assessed at 70.1% (*n* = 1953/2787) of visits and interval growth for children under 6 months was assessed at 70.4% (*n* = 1168/2431) of visits. Child development activities were assessed less frequently; general child development counseling was provided 52.1% of the time and availability of playthings for stimulation in the home was assessed at 6.6% of visits. Adjusted age for preterm infants was assessed 21.6% (*n* = 232/1074) of the time for those children under age 24 months with a documented gestational age recorded.

**Table 4:** Progress of Pediatric Development Clinics services in the first 24 months of implementation

Time periods	0–6 months		7–12 months		13–18 months		19–24 months		
	n	%	n	%	n	%	n	%	*P* value*
Total number of visits during period	315	11.3	716	25.7	890	31.9	866	31.1	
Visit location (*N* = 2787)									
Rwinkwavu District Hospital	250	79.4	363	50.7	391	43.9	444	51.3	<0.001
Decentralized Health Center	65	20.6	353	49.3	499	56.1	422	48.7	
Danger signs assessed at visit (*N* = 2787)									
Yes	136	43.2	508	71.0	684	76.9	625	72.2	<0.001
No	179	56.8	208	29.1	206	23.2	241	27.8	
Age recorded at visit (*N* = 2529)									
Yes	272	98.2	617	97.8	795	98.4	802	98.7	0.302
No	5	1.8	14	2.2	13	1.6	11	1.4	
Adjusted age for preterm babies ≤24 months (*N* = 1074)									
Yes	27	20.8	91	31.1	64	18.2	50	16.7	0.003
No	103	79.2	202	68.9	288	81.8	249	83.3	
Weight recorded at visit (*N* = 2787)									
Yes	312	99.1	708	98.9	876	98.4	859	99.2	0.790
No	3	1.0	8	1.1	14	1.6	7	0.8	
Height recorded at visit (*N* = 2787)									
Yes	308	97.8	695	97.1	877	98.5	856	98.9	0.021
No	7	2.2	21	2.9	13	1.5	10	1.2	
Head circumference recorded at visit if ≤18 months (*N* = 2431)									
Yes	284	96.3	607	92.1	779	97.4	696	97.3	<0.001
No	11	3.7	52	7.9	21	2.6	19	2.7	
Interval growth calculated at visit if ≤6 months (*N* = 1659)									
Yes	196	74.5	385	76.4	351	73.1	236	57.3	<0.001
No	67	25.5	119	23.6	129	26.9	176	42.7	
Vaccination status recorded (*N* = 2529)									
Yes	259	93.5	553	87.6	696	86.1	721	88.7	0.171
No	18	6.5	78	12.4	112	13.9	92	11.3	
Feeding difficulty assessed (*N* = 2529)									
Yes	244	88.1	496	78.6	664	82.2	734	90.3	<0.001
No	33	11.9	135	21.4	144	17.8	79	9.7	
General child development counseling received (*N* = 2675)									
Yes	64	21.8	256	38.2	519	59.9	554	65.6	<0.001
No	229	78.2	415	61.9	347	40.1	291	34.4	
Playthings and stimulating activities in the home assessed (*N* = 2529)									
Yes	7	2.5	32	5.1	58	7.2	69	8.5	<0.001
No	270	97.5	599	94.9	750	92.8	744	91.5	
Next rendez-vous recorded (*N* = 2787)									
Yes	309	98.1	694	96.9	869	97.6	841	97.1	0.658
No	6	1.9	22	3.1	21	2.4	25	2.9	

*P-values were calculated using a non-parametric test for trend.

Significant, steady improvements in assessment over time were documented in development counseling – from 21.8% (*n* = 64/2675) in the first 6 months to 65.6% (*n* = 554/2675) in the most recent 6 months of clinic implementation (*p* < 0.001) as well as evaluation of home stimulation activities from 2.5% (*n* = 7/2529) to 8.5% (*n* = 69/2529, *p* = 0.001). Danger signs assessment improved substantially between the first and second 6 months from 43.2% (*n* = 136/2787) to 72.2% (*n* = 625/2787), then stabilized (*p* < 0.001). Feeding difficulty and vaccination status assessments were more varied across time: feeding difficulty fluctuated from 88.1% (*n* = 244/2529) in the first 6 months to 78.6% (*n* = 496/2529) then 82.2% (*n* = 664/2529) and up to 90.3% (*n* = 734/2529) in the most recent 6 months of implementation (*p* < 0.001). Vaccination status also varied, with some declines from 93.5% (*n* = 259/2529) in the first 6 months of implementation to 87.6% (*n* = 553/2529), then 86.1% (*n* = 696/2529) and 88.7% (*n* = 721/2529, *p* = 0.171). Proportion of interval growth assessment dropped significantly in the last 6 months from 74.5% (*n* = 196/1659) to 57.3% (*n* = 236/1659, *p* < 0.001).

## Discussion

We report the successful implementation of the novel PDC in a rural African setting during its first 24 months of operation. Our findings show relative completeness of follow-up visits according to PDC protocol for 2 years in this population of previously underserved high-risk children. In a setting with very few specialists, the program was designed to tackle a health worker shortage based on a task shifting model that relies primarily on nurses and social workers to deliver care with general practitioner oversight. Our findings show this task shifted model is feasible for delivering follow-up care for the early identification of complications among a population of children at risk of medical, nutritional, and developmental impairments. This builds on the large body of evidence demonstrating successes of task shifting in other domains, especially in HIV care [30].

The PDC is reaching a diverse population of at-risk children under-5 years of age, many of whom were previously not reached at all [31]. By providing services for these high-risk children, PDC has the potential to empower caretakers who face stigma and high levels of stress when looking after children experiencing developmental problems [32]. The targeted population of PDC was focused on those infants born with perinatal risk whose long-term outcomes could benefit significantly with early assessment and intervention to catch up with their non-medically vulnerable peers– namely, those born preterm and/or low birth weight and significant HIE, as well as other less common perinatal conditions requiring medical and nutritional follow-up. The majority of children were less than 6 months at the time of enrollment, which is critical for ensuring services are provided as early as possible. By enrolling children in their first few months of life and routinely screening these children for medical, nutritional and developmental concerns, the PDC has created a platform for early identification and intervention rather than the default system of waiting until children are sick and present to care before care is provided [33]. Additionally, creating a comprehensive platform rather than a vertical program targeted solely at one population, such as preterm infants for KMC, optimizes the impact of investing in a chronic care operational delivery model.

Most caretakers were mothers and were married or cohabiting. Caretakers play an enormous role in the development of young children and the very rare involvement of father’s in the program is an aspect that the clinic is trying to address. Involving fathers in children’s care can mean not only that when mothers are not available, they can step in and avoid missing appointments, but more importantly, that fathers are also involved in early stimulation activities at home [34] which can improve children’s developmental outcome [35]. Additionally, supporting older siblings who may serve as caretakers is an area for consideration in future programming. Further, almost half of caretakers were less than 25 years of age, with several adolescent mothers, which has been shown to be associated with social and medical vulnerability and a barrier to adherence to care [36]. This unique population of mothers can benefit from services promoting positive parenting and stimulation [37] to empower them to create a nurturing home environment for optimal development.

In serving this unique and vulnerable population of patients, the provision of conditional food and cash transfers aims to break barriers to adherence to chronic care and ensure adequacy of access to at least minimum nutritional needs. As the PDC patients’ families were primarily not eligible for government support, social support is an integral component of the PDC program. The majority of patients in the program benefit from some form of conditional food and cash transfers, particularly transport fees to reduce financial barriers to accessing the PDC. Future research to assess PDC impact on patient outcomes is needed, which, in combination with a costing analysis – that takes into consideration required start-up costs for equipment and staff training, staff time, social supports, medications, supplies, operational costs, as well as costs of mentorship and supervision for both health center and hospital teams – will be important to assessing potential for scale-up.

Although there was significant variability in the proportion of visit assessments completed over time, completion of screening for danger signs and developmental counselling improved significantly. Possible explanations for this finding are that over time, providers became more familiar and more comfortable with assessing these components. Additionally, mentorship visits were conducted at least monthly by the hospital staff to health center clinics where the district hospital PDC team provided practice-based guidance on care protocols, referral processes, and nutrition and development counseling to health center teams. These visits have likely contributed to this improvement. Percent complete recording of adjusted age, although improved since the first 6 months of PDC, have fluctuated over time. Interval growth calculation was steadily above 70% until the last 6 months, when we saw a sharp reduction in percent complete recording. These quality gaps highlight the particular challenges of growth monitoring in preterm infants where growth standards are unique and gestational age is often not known. We found that variables requiring calculation, such as adjusted age for preterm infants and interval growth calculation, were completed less frequently than those variables requiring simple recording, such as age, weight and head circumference. These well-done components are also captured in other clinics, such as the Integrated Management of Childhood Illness, and clinicians are accustomed to recording them. Although a clinician’s ability to calculate interval growth will be impacted if the weight of the previous visit was not recorded in patient charts, the fact that weight was recorded more than 95% of the time makes this less likely to be an issue. Qualitative exploration of factors leading to the reduction in recording are needed to understand these findings. Automated calculation tools, such as job aids or mHealth tools, could facilitate the completion of these assessments, provide clinical reminders, and video teaching and counseling supports, as has been successfully done in other task-shifted programs [38, 39].

More than the half of PDC children who were eligible for ASQ had one completed. The integration of developmental screening has proven challenging as nurses have to read questions to caretakers due to low literacy and it takes time to administer, which can be particularly burdensome as patient volume increases. Furthermore, the ASQ-3 was a new tool in our setting and nurses from PDCs needed time to become familiar with the process. Further efforts are needed to be able to appropriately translate the findings of a developmental screening into direct intervention in settings such as Rwanda where no locally validated tools exist [18]. Some promising tools have emerged that were designed specifically for easy assessment and intervention in low- and middle-income countries including the International Guide for Monitoring Child Development [40] and the Caregiver-Reported Early Childhood Development Index (CREDI) [41], which could be more appropriate but require additional study for feasibility in the PDC. In the meantime, a play and communication module has been introduced more recently into the protocol and provider training in order to help boost the developmental assessment and intervention skills of PDC providers.

This study has some limitations. Because we used routinely-collected data from patient charts entered into EMR, data were missing or incomplete for several variables. However, we believe the data available gives an accurate description of the clinic’s patients and services. In addition, when possible, we verified discrepant data in patient charts and in the EMR. Another limitation could be the generalizability of the study findings to other settings, as the pilot for PDC was conducted in rural southern Kayonza District in Rwinkwavu District Hospital catchment area which has received support from Partners In Health/Inshuti Mu Buzima since 2005.

## Conclusion

Our study demonstrates that the PDC model is a feasible design that supports early identification and intervention for medical, nutritional, and developmental complications among high-risk children in a rural African setting. Using a task-shifting model, nurses and social workers were able to follow a standardized protocol for patient visits; however as a new program there are areas for ongoing improvement in the quality of care. Future research is needed to understand the outcomes of the children benefiting from the PDC program as well as costs of PDC implementation.
